# Heparin-Modified Amniotic Membrane Combined With Growth Factors for Promoting Corneal Wound Healing After Alkali Burn

**DOI:** 10.3389/fbioe.2020.599800

**Published:** 2020-11-23

**Authors:** Xuan Zhao, Xin Zuo, Jing Zhong, Bowen Wang, Saiqun Li, Yichen Xiao, Jin Yuan

**Affiliations:** State Key Laboratory of Ophthalmology, Zhongshan Ophthalmic Center, Sun Yat-sen University, Guangzhou, China

**Keywords:** amniotic membrane, heparin, sustained release, epithelial growth factor, corneal alkali burns

## Abstract

Ocular chemical burns are potentially blinding ocular injuries and require urgent management. Amniotic membrane (AM) transplantation is an effective surgical treatment, one of the reasons is because AM is a rich source of growth factors that can promote epithelialization and wound healing. However, growth factors will be gradually lost and insufficient after preparation process and long-time storage, leading to unsatisfactory therapeutic effects. Herein, we present a modified AM (AM-HEP) for the supplement and sustained release of growth factor by surface grafting heparin for treatment of ocular chemical burns. Heparin grafting rate and stability, microstructure, physical property, and sustained release of epithelial growth factor (EGF) of AM-HEP were characterized. Biocompatibility and ability to promote corneal epithelial cell growth and migration were evaluated and compared with a biological amnion, which is available on the market *in vitro*. The therapeutic effects of AM-HEP combined with EGF (AM-HEP@EGF) *in vivo* had been evaluated in a model of mouse corneal alkali burn. The results indicated that heparin was introduced into AM and maintain stability over 3 weeks at 37°C. The modification process of AM-HEP did not affect microstructure and physical property after comparing with non-modified AM. EGF could be combined quickly and effectively with AM-HEP; the sustained release could last for more than 14 days. AM-HEP@EGF could significantly promote corneal epithelial cell growth and migration, compared with non-modified AM and control group. Faster corneal epithelialization was observed with the transplantation of AM-HEP@EGF *in vivo*, compared with the untreated control group. The corneas in the AM-HEP@EGF group have less inflammation and were more transparent than those in the control group. The results from *in vitro* and *in vivo* experiments demonstrated that AM-HEP@EGF could significantly enhance the therapeutic effects. Taken together, AM-HEP@EGF is exhibited to be a potent clinical application in corneal alkali burns through accelerating corneal epithelial wound healing.

## Introduction

Cornea is located on the outermost layer of the eye and is easily injured (Meek and Knupp, 2015). Ocular chemical burns are responsible for 11.5–22.1% of ocular injuries (Sharma et al., 2018). Alkali burn is the most common among chemical burns and considered as ophthalmic emergencies, which require urgent management (Reim et al., 2001; Welling et al., 2016). Amniotic membrane (AM) is the innermost layer of the placenta and protects a fetus during pregnancy. It was reported that AM was first used to treat ocular burns in 1940, and it was not widely used until people began to use it for conjunctival tissue replacement in the 1990s (Dua et al., 2004). Currently, AM products were approved by the Food and Drug Administration (e.g., Prokera, Amniograft and Ruiji biological AM) that are clinically used to help the ocular surface reconstruction due to its unique biological characteristics (Palchesko et al., 2018). AM transplantation is an effective medical therapy for corneal alkali burn due to its outstanding biological performances (Maharajan et al., 2007; Singh et al., 2013). As a temporary covering material, AM can promote corneal epithelialization and inhibit inflammation (Kobayashi et al., 2003; Liang et al., 2012; Sharma et al., 2015). To some extent, it alleviates the shortage of corneal donors and has become a usual biomaterial in ophthalmic surgery (Fan et al., 2016). The biological performances of AM are due to various growth factors from amniotic epithelial cells (Brodovsky et al., 2000; Sridhar et al., 2000; Joseph et al., 2001). However, growth factors will be gradually lost and insufficient and cannot maintain the activity after AM preparation process and long-time storage (Pereira et al., 2016). Usually, AM needs pretreatment before use. These processes may include inactivation of bacteria and viruses, freeze drying, etc. Besides pretreatment, AM may also need to be stored and transported if it is not used immediately or used in another place. At present, the clinical use of AM is mainly divided into three types: fresh AM, freeze-dried AM, and acellular AM (Sharma et al., 2016; Bandeira et al., 2019; Zhou et al., 2019; Schuerch et al., 2020). The advantage of fresh AM is the growth factors is abundant, so it has more significant effects in anti-inflammatory, inhibiting scar formation, inhibiting neovascularization, and promoting epithelial cell proliferation (Chugh et al., 2015; Sahay et al., 2019). However, its storage time is short, which limits the production and clinical application. Freeze-dried AM can be stored for a longer period at room temperature. Compared with fresh AM, this method prolongs the preservation time, but the growth factors in freeze-dried AM still have a great loss (Paolin et al., 2016). In addition, freeze-drying has potential damage to the microstructure of AM, affecting the mechanical strength after rehydration and limiting some applications that require high mechanical strength (Paolin et al., 2016; Palchesko et al., 2018). The main component of acellular AM is collagen, and the content of growth factors in the matrix layer is low (Lai and Ma, 2013). Therefore, acellular AM is more often used as a carrier for cell culture (Salah et al., 2018; Abazari et al., 2019). The existing methods for preparing AM cannot balance storage time and activity. Whether it is stored in a dry state or in a wet state, it is difficult to keep growth factors active for a long time in the same storage environment as AM. These may lead to unsatisfactory therapeutic effects.

Growth factors are important regulators that stimulate cell growth, proliferation, migration, differentiation, adhesion, and ECM deposition in wound healing (Lu et al., 2001; Yu et al., 2010). Epithelial growth factor (EGF) is a well-known potent mitogen and major pathway that initiates migration and proliferation for corneal epithelial cells (Noh, 2012; Wang et al., 2013). It plays a key role in epithelial reconstruction after ocular surface injury. Hence, EGF is considered beneficial to the recovery of corneal alkali burn. Supplementing growth factor becomes an effective method to solve this problem. AM has abundant collagen fibers and reticular fibers, which can load and release drug or growth factors (Di Lullo et al., 2001; Denis et al., 2005). Therefore, growth factors can be supplemented by soaking AM in solution before use to adsorb growth factor. However, AM has limited loading capacity for growth factors, which cannot meet the demands for treatment. A more efficient way to load growth factors is a desiderate problem needed to be solved. Heparin is a sulfated mucopolysaccharide, a natural anticoagulant substance in animals, and has excellent cell compatibility (Aslani et al., 2020). Heparin has been widely used in clinic such as thromboembolic diseases, hemodialysis, and cardiovascular surgery (Ayerst et al., 2017; Aslani et al., 2020; Yu et al., 2020). With the advancement of pharmacology and clinical medicine, the application of heparin continues to expand (Chistolini et al., 2020). The negatively charged sulfate groups of heparin can bind and release various positively charged growth factors such as transforming growth factor β (TGF-β) and vascular endothelial growth factor (VEGF) (Jo et al., 2015). It may be an effective way to supplement growth factors. But few researchers have focused on heparin-modified AM combined with growth factors for corneal alkali burn treatment.

**FIGURE 1:**
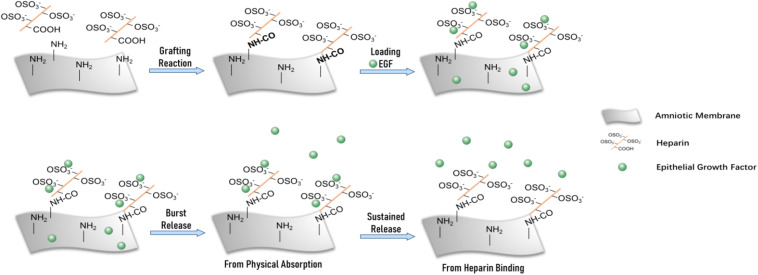
Schematic of modified amniotic membrane preparation, EGF adsorption, and release on AM-HEP.

In this study, we used AM as a substrate material and surface graft heparin to combine with EGF for achievement of supplement and sustained release (Figure 1). The physicochemical properties, microstructure, and EGF sustained release of modified AM (AM-HEP) were characterized. Biocompatibility and ability to promote corneal epithelial cell growth and migration were evaluated *in vitro*. The therapeutic effects of AM-HEP *in vivo* were evaluated in mouse corneal alkali burn model.

## Materials and Methods

### Materials

Amniotic membrane was collected in accordance with the tenets of the Declaration of Helsinki from a woman who had a healthy pregnancy after cesarean birth; informed consent was obtained. It complies with the relevant provisions of the Regulations on the Administration of Medical Institutions promulgated by the State Council of the People’s Republic of China, and the selection of materials during the research conforms to ethical standards. Biological amnion (BAM) was purchased from JiXi RuiJi BioTechnology Co., Ltd., (Jiangxi, China). Heparin sodium salt (HEP) and methylene blue were obtained from Ryon Biological Technology (Shanghai, China). GL Biochem Ltd., (Shanghai, China) supplied 1-ethyl-3-(3-dimethylaminopropyl) carbodiimide (EDC) and *N*-hydroxysuccinimide (NHS). 2-Morpholinoethane sulfonic acid (MES) buffer (0.05 M, pH 5.5) was purchased from Leagene Biotech (Beijing, China). Deionized water was obtained from the water purification system (Millipore S. A. S, France). Murine EGFs were obtained from PeproTech (Rocky Hill, NJ, United States). All cell culture–related reagents were purchased from Sigma–Aldrich (St. Louis, MO, United States). Male and female C57 mice (6–8 weeks old) (Beijing Vital River Laboratory Animal Technology Co., Ltd., Beijing, China) were used as animal transplant recipients. Their use was approved by the Medicine Ethics Committee at Sun Yat-sen University, Guangzhou, China.

### Heparinization of Amniotic Membrane

Heparin sulfate salt (1 mg/mL) was dissolved in 0.05 M MES buffer (pH 5.5). EDC and NHS were dissolved in deionized water at 4°C, respectively (15 mg/mL). Then, the three solutions were mixed with a mass ratio of HEP:EDC:NHS = 1:5:4 at 4°C. AM was soaked in this mixture and stirred by using the rotor-magnetic stirring at 4°C for 12 h. Then AM was rinsed three times with deionized water and dried under constant temperature (30°C) and humidity (50%).

### Heparin Content Characterization

Heparin content was determined by a UV3802 ultraviolet-visible spectrophotometer (Shanghai UNICO, Shanghai, China) after combination with methylene blue. The scans were registered from 500 to 800 nm. The detection wavelength of heparin was determined after determining the characteristic absorption peak of heparin–methylene blue complex. Then, the right amount of heparin was diluted with deionized water to obtain the final solution concentrations of 1, 2, 3, 4, 6, 8, and 40 μg mL^–1^ and mixed with a volume ratio of heparin solution to 0.05% methylene blue solution = 25:1. The absorbance was measured at the detection wavelength, and the mass concentration was used to draw a standard curve of the absorbance. Heparin content of the mixture after reaction was determined to measure the heparin content grafted on AM. AM-HEP was immersed in 10 mL phosphate-buffered saline (PBS) (pH 7.4) and constantly shaken in a shaker at 37°C. The extract liquid was collected at regular time intervals (1, 3, 5, 7, 9, 11, 14, 17, and 21 days). The volume of the medium was kept constant by adding the same amount of PBS. The heparin content of extract liquid was determined to evaluate the residual heparin content on AM-HEP.

### Fourier Transform Infrared Spectroscopy

The infrared structures of AM and AM-HEP were analyzed using Fourier transform infrared (FTIR) spectroscopy (VERTEX 70, Bruker, Germany). Before acquiring an FTIR spectrum of a sample, a background spectrum was collected. All the spectra were obtained from 700 to 4,000 cm^–1^.

### Microscopic Morphology Assays

Samples were mounted on aluminum stubs and sputter coated with platinum for 70 s before examination by scanning electron microscope (EVO18; Zeiss, Oberkochen, Germany).

### Characterization of Physical Properties

Light transmission was measured with a UV3802 ultraviolet visible spectrophotometer (Shanghai UNICO, Shanghai, China) at 37°C over a range from 400 to 800 nm. Before testing, samples were immersed in deionized water for 1 h to absorb water completely, and the samples were then cut into rectangles. Thickness was measured with a thickness gauge. The experiments of thickness characterization were carried out in quintuplicate. For water content characterization, the samples were immersed in deionized water for a specific time period. After being quickly blotted with filter paper to remove superficial water, the weights of the wet samples (M_t_) were measured with electronic scales. Then, the samples were vacuum dried to a constant weight (M_0_). The water content of the samples was calculated according to the following equation: *W*_t_ = (*M*_t_ – *M*_0_)/*M*_t_ × 100%. Every reported value was an average of at least five measurements. The tensile strength was determined with a uniaxial load testing instrument (Model #5567; Instron Corporation, Issaquah, WA, United States), equipped with a load cell of 10 N capacity at a crosshead speed of 10 mm min^–1^ and an initial grip separation of 10 mm. The deionized water equilibrated samples were cut into dumbbell-shaped specimens of identical rectangular gauge areas (width: 5 mm, gauge length: 10 mm) with two 8-mm end tabs. To avoid the breakage and slippage of samples in the jaws, there were 8-mm-wide tabs on the end of each dumbbell. Samples were not stress preconditioned before testing to failure. During the tests, the samples were hydrated. Every reported value is an average of at least five measurements.

### *In vitro* Load and Release of EGF

Samples were placed in an EGF solution (1 μg mL^–1^) at 25°C for regular time intervals (1, 2, 3, and 4 h), which allowed EGF to combined with AM-HEP and AM. EGF concentration in the solution after soaking was determined by enzyme-linked immunosorbent assay (ELISA) kit (Neobioscience Technology Co., Ltd., China) to measure the capacity of AM-HEP to EGF. To obtain the release profiles, samples were placed in 10 mL PBS containing 0.1% bovine serum albumin and in a constant temperature shaking water bath [60 rpm, 37°C; Zhong Yi Guo Ke (Beijing) Technology Co., Ltd., China] for 14 days. Samples of medium were taken at prescribed times and determined by ELISA. An equivalent volume of fresh medium was added after taken. Optical densities (ODs) were measured at 450 nm using a microplate reader.

### *In vitro* Cell Culture, Biocompatibility and Wound Healing Assay

The mouse corneal epithelial cells (CECs) were obtained from fresh cornea and cultured in Dulbecco modified eagle medium Nutrient Mixture F-12 (Gibco BRL, Waltham, MA, United States) with 10% fetal bovine serum (Gibco), 100 U mL^–1^ penicillin, and 100 μg mL^–1^ streptomycin (Gibco). Cells were grown to confluency in 25-cm^2^ polystyrene tissue culture flasks at 37°C in 5% CO_2_ and 95% air, and confluent cells were subcultured every 2–3 days by trypsinization with trypsin/EDTA solution.

To characterize the proliferation of CECs on tissue culture plates and the samples, AM and AM-HEP were placed in 24-well tissue culture plates (BD, Japan), and CECs were seeded onto the surface of the samples (experimental group, *n* = 10) or tissue culture plates at 5,000 cells per cm^2^. The proliferation of the CECs on the films was quantitatively determined by CCK-8 assay at the OD value of 450 nm with a microplate reader. OD value was recorded at 1 and 3 days.

To observe the morphology of CECs on tissue culture plates, AM and AM-HEP samples were washed three times in PBS under aseptic conditions, sterilized by ultraviolet radiation for 2 h. After that, the samples were transferred to a 24-well tissue culture plate. A certain volume of CECs suspension was seeded onto the samples separately at 5,000 cells per cm^2^. The culture medium was replaced every 2 days. Microscopic photographs were taken by an inverted fluorescence microscope (Zeiss Observer A1) for observing the cellular morphology.

Sterile AM-HEP was soaked in EGF solution (1 μg mL^–1^) for 3 h before use. Proliferation and migration promotion of BAM and AM-HEP combined with EGF (AM-HEP@EGF) proceeded by coculture with CECs after CECs were seeded on 24-well tissue culture plates at 5,000 cells per cm^2^ for 12 h. After CECs were grown to confluency, a scratch was made through the confluent cells with a 200 μL pipette tip and cultured under serum-free conditions. Micrographs were taken at determined times with an inverted fluorescence microscope (Zeiss Observer A1). The healing rate was calculated according to the following equation: healing rate = (*W*_t_ – *W*_0_)/*W*_t_ × 100%, where *W*_t_ is the width of scratch measured at regular time, and *W*_0_ is the width of scratch made by pipette tip at the beginning of the experiment.

### Corneal Alkali Burn Model in Mouse

C57 mice (half males and half females) were housed under specific pathogen-free conditions and used as animal transplant models. All animals were treated in accordance with the Association of Vision and Ophthalmology Statement on the Use of Animals in Ophthalmic and Vision Research. Thirty mice were divided into three groups, with 10 mice in each group. A 2-mm-diameter filter paper was soaked in 0.4 M NaOH solution for 60 s and then placed it to the center of the mouse cornea for 60 s to produce a corneal burn. After removing the filter paper, the cornea was rinsed with 20 mL saline for 60 s. AM and AM-HEP were trephined into 3 mm in diameter and then washed in sterile PBS. After the implants were dried, they were sealed with a sealing machine in an aseptic environment. Before the surgery, AM-HEP was immersed in EGF solution (1 μg/mL) for 4 h to absorb water and EGF, while AM was immersed in PBS for the same time. Samples were implanted into the right cornea of the mouse by covering. Operations were performed on only one eye of each animal. Corneas without AM-HEP@EGF after molding were used as a control group. Follow-up clinical examinations were performed at 1, 3, 7, and 14 days after injury, including sodium fluorescein staining to assess epithelial integrity, slit lamp microscopy to assess corneal optical clarity, corneal deformation assessments, and rejection reaction determinations. All animal experiments were performed with permission from the Medicine Ethics Committee at Sun Yat-sen University, China.

## Clinical Evaluations

Corneal opacity was scored from 0 to 4 with slit-lamp at 1, 3, 7, and 14 days, using the following grading system: 0 (no opacity, completely clear cornea), 1 (slightly opaque, iris and lens visible), 2 (moderately opaque, iris and pupils still detectable), 3 (severely opaque, pupils hardly visible), and 4 (completely opaque, pupils invisible).

Quantitative analysis for the wound healing rate of corneal epithelium was measured using ImageJ software and calculated with the following formula: healing rate = (*S*/*S*0) × 100%, where *S*0 represent the corneal alkali burn areas of experimental and model animals, *S* represent the corneal epithelial defect area of experimental and model animals.

### RNA Isolation and Quantitative Real-Time Polymerase Chain Reaction

We used quantitative real-time polymerase chain reaction (qRT-PCR) to detect inflammatory-related genes [interleukin 1β (IL-1β), IL-6, tumor necrosis factor α (TNF-α)] *in vivo* at 3, 7, and 14 days after operation. The mice were sacrificed by cervical dislocation. The total RNA was extracted from cornea tissue of experimental and model animals with TRIzol (Invitrogen). The quantity and quality of the isolated RNA were determined with a NanoDrop2000 spectrophotometer (Thermo Scientific). RNA was reverse-transcribed into cDNA with a PrimeScript RT reagent kit with gDNA Eraser (TaKaRa Biotechnology, Japan), according to the manufacturer’s protocol. Finally, RT-PCR analysis was performed with an SYBR Green System (GeneCopoeia) on an RT-PCR instrument (QuantStudio 6 Flex, Life Technologies). cDNA samples were amplified at 50°C for 2 min and 95°C for 10 min, with 40 cycles at 95°C for 15 s, 60°C for 30 s, and 72°C for 30 s. The relative quantification of target genes was performed through normalization to glyceraldehyde-3-phosphate dehydrogenase (GAPDH), and the 2^–ΔCt^ method was used to calculate the gene expression. The PCR primer sequences were as follows. For GAPDH: forward primer: AATGGATTTGGACGCATTGGT; reverse primer: TTTGCACTGGTACGTGTTGAT; for IL-1β: forward primer: AATTACCTGCTCATCTTCGGAGT; reverse primer: TCCATAGGAGAGGCTGAGATTC; for IL-6: forward primer: GGCGGATCGGATGTTGTGAT; reverse primer: GGACCCCAGACAATCGGTTG; for TNF-α: forward primer: GGAACACGTCGTGGGATAATG; reverse primer: GGCAGACTTTGGATGCTTCTT.

### Histology

Hematoxylin–eosin sections were made to examine the recovery of corneal epithelium and stroma. At 14 days after the transplantation, the mice were sacrificed by cervical dislocation. The corneas were excised from each mouse and fixed for 24 h in a 10% neutral-buffered formalin solution at room temperature. They were then cut, stained with hematoxylin–eosin, and observed under an optical microscope (Aperio CS2, Leica, Germany).

### Statistical Analysis

All data are expressed as means ± standard deviation. Statistical analyses were performed using GraphPad (GraphPad Software, United States). Student *t* test was used to compare differences between groups. Statistical significance was defined as *p* < 0.05.

## Results

### Heparinization of Amniotic Membranes

**FIGURE 2:**
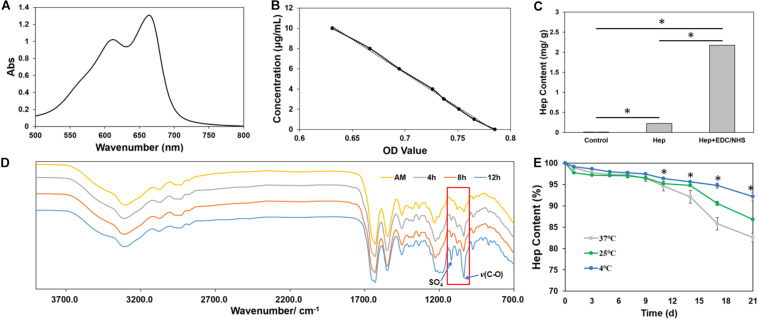
Characterization of grafted heparin onto AM. **(A)** UV absorption curve of heparin combined with methylene blue from 500 to 800 nm. **(B)** Concentration–absorbance standard curve of heparin combined with methylene blue. **(C)** The amount of grafted heparin on amniotic membrane. **(D)** Infrared spectra of amniotic membrane grafted with heparin. **(E)** Time-dependent changes of heparin content in AM at different temperatures. ^∗^Statistically significant at *p* ≤ 0.05.

The quantity of heparin grafted onto AM was assessed by using ultraviolet spectrum and FITR. The ultraviolet spectrum of heparin–methylene blue from 500 to 800 nm was scanned, and the maximum absorption peak was observed at 662 nm (Figure 2A). Absorbance–concentration standard curve was established for quantitative detection (Figure 2B). Heparin content of AM-HEP was much higher after chemical modification than that of AM, which was only soaked in heparin solution for the same time and non-modified AM (Figure 2C). To further determine the graft was effective, FITR of AM after a different modification time proceeded (Figure 2D). With the increase in modification time, absorption at 1,035 cm^–1^ [v(C-O)] and that at 1,120 cm^–1^ (SO_4_) were obviously increased. This is because introduction of heparin brought more C-O stretching and sulfate radical. These results indicated heparin was successfully grafted onto AM and in a time-dependent manner. Then heparin graft stability was detected under different temperature (Figure 2E). The results showed that lower storage temperature (4°C) was a benefit to keep the heparin content. Although high environment temperature could lead to more heparin lost, approximately only 15% heparin was released from AM-HEP under body temperature (37°C) at 21 days. There was a stable combination between heparin and AM.

### Characterization of Modified Amniotic Membranes

**FIGURE 3:**
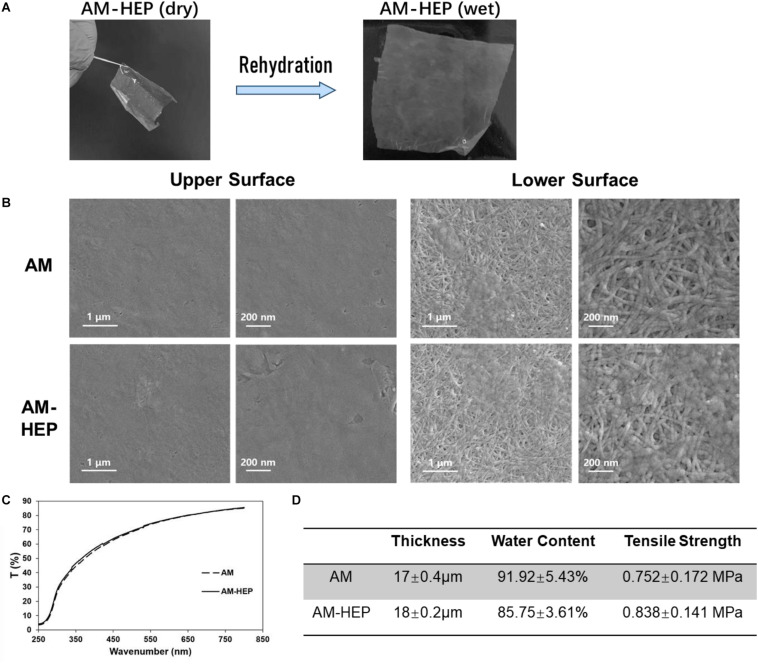
Comparison of physical properties before and after AM modification. **(A)** Modified AM can restore the macro morphology of natural AM by rehydration. **(B)** Transmittance of AM before and after modification. **(C)** Thickness, water content, and tensile strength of AM before and after modification. **(D)** Scanning electron micrographs of the upper and lower surfaces of AM before and after modification.

AM-HEP was dehydrated for storage after modification, and it can take up water quickly before use. After rehydration, AM-HEP had a similar macro morphology to fresh AM (Figure 3A). The upper and lower surface microstructures of AM and AM-HEP were observed (Figure 3B). Basilar membrane and compact layer of AM were retained; the structure of collagen fiber kept the integrity and was still clear. In order to determine whether the physical properties related to the ocular surface application have changed after modification, light transmittance, thickness, water content, and mechanical strength of AM and AM-HEP were characterized (Figures 3C,D). The transmittances of AM and AM-HEP showed no significant difference under visible light. The light transmission values of the samples increased with the increase in wavelength and can reach nearly 90%. AM and AM-HEP had a similar thickness; indicated AM-HEP was still a thin film that is easy to apply to the ocular surface. Water content of AM-HEP was decreased, while tensile strength of AM-HEP was increased after modification compared with that of AM. The higher tensile strength of AM-HEP can promote the operability in surgery and nursing. These results indicated the modification process would not change the structure and property of AM mainly.

### EGF Load and Release

**FIGURE 4:**
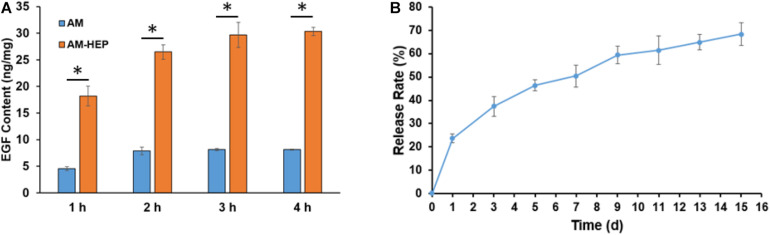
EGF load capacity of AM and AM-HEP and release of EGF from AM-HEP@EGF. **(A)** EGF content of AM and AM-HEP after soaking EGF solution for 1, 2, 3, and 4 h. **(B)** Cumulative release of EGF from AM-HEP@EGF. ^∗^Statistically significant at *p* ≤ 0.05.

Load and release of EGF results showed that AM-HEP had better EGF load capacity than AM, fast EGF adsorption, and sustained release effect (Figures 4A,B). AM-HEP can combine with EGF quickly, and the content can reach 30 μg/mg after soaking into EGF solution for 3–4 h. AM-HEP@EGF, which was soaked for 4 h in EGF solution, was used in follow-up experiments. The release curve exhibits an exponential tendency. The burst release of EGF from AM-HEP@EGF in the first day is about 25% of the total EGF loading amount, and that is followed by a slower release. The sustained release of EGF from AM-HEP@EGF takes more than 2 weeks. The total EGF released from AM-HEP@EGF has exceeded 60% on the 14th day.

### *In vitro* Biocompatibility

**FIGURE 5:**
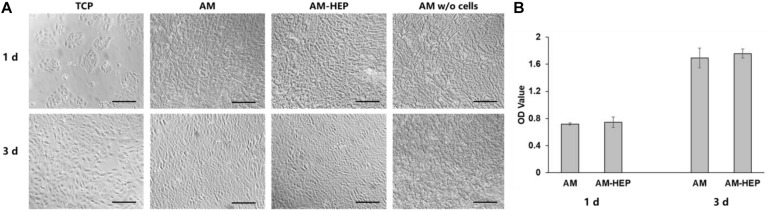
The proliferation of CECs on AM and AM-HEP. **(A)** Morphology of CECs seeded on TCP, AM, and AM-HEP. Scale bar = 50 μm. **(B)** Proliferation experiment of AM and AM-HEP.

Morphologies of CECs on AM and AM-HEP at 1 and 3 days were observed (Figure 5A). The cells could attach onto AM and AM-HEP at 1 day, and they were almost completely covered by CECs after 3 days. AM without cells seeded on it was shown to see cells on samples more exact because fiber structure of AM may obstruct the observation. The CCK-8 test showed the proliferation of CECs on AM and AM-HEP (Figure 5B). Compared with AM, AM-HEP also had good cytocompatibility. CECs had a similar growth rate on the days tested. There results showed AM-HEP maintained excellent biocompatibility after the modification.

### Cell Proliferation Assay

**FIGURE 6:**
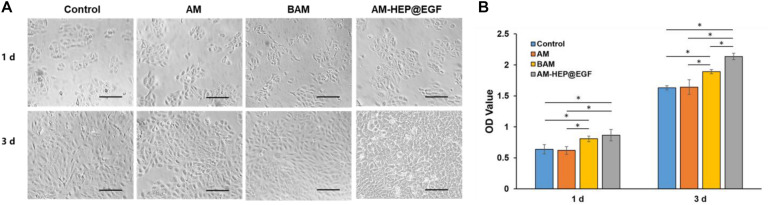
The effects of AM-HEP@EGF on CECs. **(A)** Morphology of CECs that coculture with AM, BAM, and AM-HEP@EGF. Scale bar = 50 μm. **(B)** The proliferation of CECs which cocultured with AM, BAM, and AM-HEP@EGF. ^∗^Statistically significant at *p* ≤ 0.05.

To investigate the proliferation-promoting effect of AM-HEP@EGF on CECs, CECs were cocultured with AM, BAM, and AM-HEP@EGF. Morphologies of CECs were captured, and CCK-8 test was proceeded (Figures 6A,B). The CECs cocultured with BAM and AM-HEP@EGF showed better proliferation on the first day, with more cell number and a tendency to be congregated. On the third day after cocultivation, the cells in the AM-HEP@EGF group had overgrown the bottom of the culture plate, and the cells were tightly connected. The CCK8 test also shows that AM-HEP@EGF can promote cell proliferation more effectively than that of BAM on the third day.

### Wound Healing Assay

**FIGURE 7:**
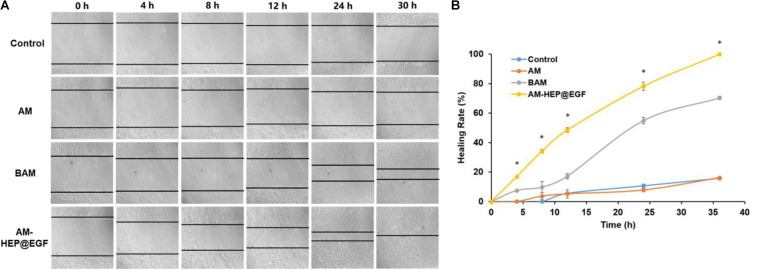
Wound healing assays of CECs cocultured with AM and AM-HEP@EGF. **(A)** Morphology of CECs that coculture with AM, BAM, and AM-HEP@EGF. **(B)** Healing rate of CECs cocultured with AM, BAM, and AM-HEP@EGF. ^∗^Statistically significant at *p* ≤ 0.05.

Corneal epithelial cells wound healing assay results of AM, BAM, and AM-HEP@EGF were proceeded (Figure 7A). Tissue culture plates were used as controls. CECs cocultured with AM-HEP@EGF exhibited complete wound healing in 30 h. Compared to control and AM groups, the healing of BAM group and that of AM-HEP@EGF group were much faster, and the AM-HEP@EGF group showed the best healing. There was no significant difference between the healing speed of control and AM, which indicated that AM without growth factor could not contribute to cell migration. Healing rate was a quantitative experiment; it also showed a clear result that AM-HEP@EGF can promote cell migration (Figure 7B).

### Treatment of Alkali Burn by AM-HEP@EGF *in vivo*

**FIGURE 8:**
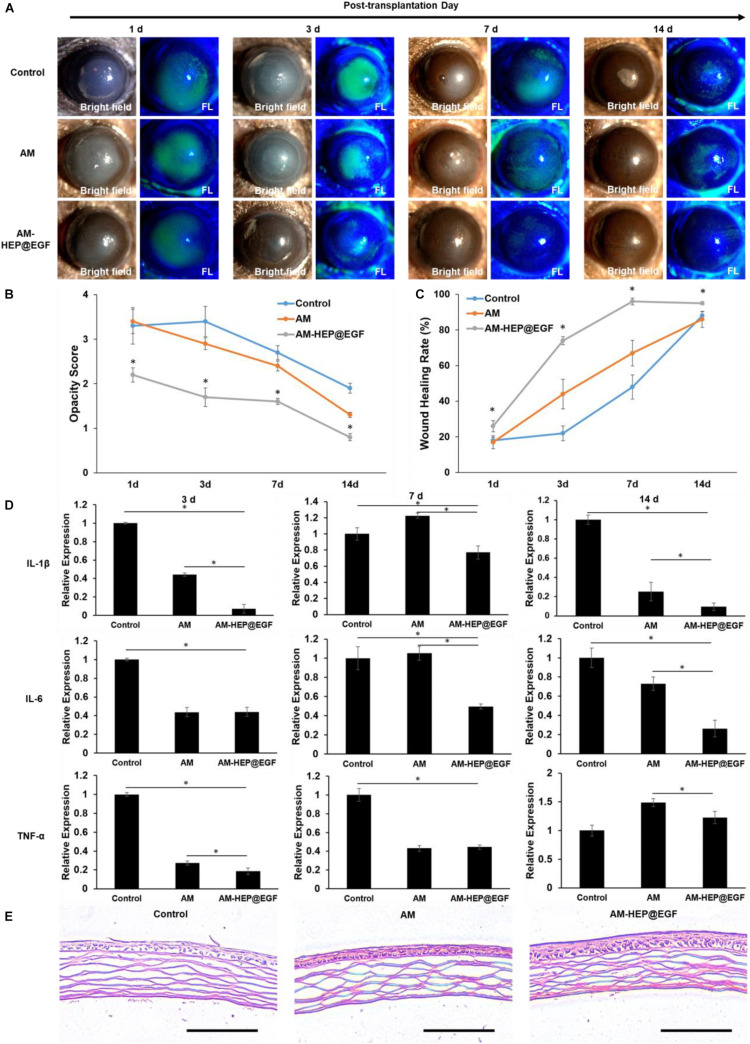
Treatment with AM-HEP@EGF induces a visible wound healing response in the cornea after alkali burn. Representative slit lamp images **(A)**, opacity score **(B)** and corneal epithelium healing rate **(C)** of untreated model (control) and AM- and AM-HEP@EGF–treated groups at 1, 3, 7, and 14 days post-operation. **(D)** qRT-PCR analysis of relative mRNA expression levels of IL-1β, IL-6, and TNF-α mRNA at 3, 7, and 14 days post-operation. **(E)** Hematoxylin–eosin staining of cornea alkali burn without treatment and treated with AM or AM-HEP@EGF at 14 days post-operation. Scale bar = 100 μm. ^∗^Statistically significant at *p* ≤ 0.05.

To further explore the function of AM-HEP@EGF, suitable size (3 mm) was made and placed onto the left eye of mice after alkali burn, and the therapeutic effects were observed under a clinical slit lamp for 14 consecutive days (Figure 8A). Compared with the control group, AM and AM-HEP@EGF groups have significant therapeutic effects on alkali burn. From days 1 through 14, the clinical scores were lower in the AM-HEP@EGF group compared to the other groups (Figure 8B). On day 1, corneas treated with AM-HEP@EGF showed higher transparency compared to the untreated group and AM-treated group. The wound healing rates that were measured according to sodium fluorescein staining results were higher in the AM-HEP@EGF group compared to the other groups (Figure 8C). In addition, no significant biodegradation of AM and AM-HEP@EGF was observed over 14 days. The mRNA expression levels of IL-1β, IL-6, and TNF-α were compared by qRT-PCR at 3, 7, and 14 days post-operation (Figure 8D). The mRNA expression levels of IL-1β and IL-6 in AM-HEP@EGF group were significantly down-regulated at 3, 7, and 14 days compared to control groups (*p* ≤ 0.05). The levels of TNF-α were significantly down-regulated at all time points expected at 14 days compared to control group (*p* > 0.05). The AM groups also down-regulated the level of IL-1β (3 and 14 days), IL-6 (3 and 14 days), and TNF-α (3 and 7 days), whereas the AM-HEP@EGF groups significantly down-regulated the level of IL-1β (3, 7, and 14 days), IL-6 (7 and 14 days), and TNF-α (3 and 14 days) compared to the AM group (*p* ≤ 0.05). Histological evaluation of the mouse cornea was performed on 14 days after treatment. The corneas were paraffin-embedded and stained with hematoxylin–eosin. The pathological differences in cornea between the control, AM, and AM-HEP@EGF groups were observed under microscope (Figure 8E). The results showed that the cornea pathology was significantly ameliorated in AM-HEP@EGF group compared to the other groups on day 14. Cornea in the AM-HEP@EGF group had a more integrated epithelial layer including squamous cells, wing cells and basal cells. These represented a promising therapeutic strategy for corneal alkali burn.

## Discussion

Many researches had focused on improvement of mechanical strength and light transmittance of AM (Konomi et al., 2013; Fan et al., 2016; Hariya et al., 2016). This is because although it has been used clinically, AM has some limitations: it lacks transparency and does not have good mechanical strength. Besides, fresh AM cannot be used immediately because of the risk of carrying bacteria and viruses. From raw materials to products, freeze-drying, cobalt-60 irradiation, and other treatment processes are required, which may reduce its growth factor content, biological activity, and therapeutic effects. Meanwhile, storage conditions and time will also affect the content of growth factors on AM. Considering that the growth factors of the AM are difficult to be stored together with AM for a long time, the most direct way to increase the growth factor content of AM is to supplement the growth factors before use. In this study, we prepared AM-HEP for the supplement and sustained release of growth factor by surface grafting heparin for treatment of ocular chemical burns.

Light transmittance and mechanical strength are important physical properties of AM for ocular surface reconstruction (Mi et al., 2010). Therefore, after verifying that heparin was successfully grafted onto the surface of the AM (Figure 2), we evaluated the performance and structure of the AM before and after modification (Figure 3). The heparin-modified AM is dehydrated. Since its main component is collagen and dry, it is conducive to its long-term storage and does not require strict low-temperature storage. After soaking, AM-HEP can quickly absorb water and return to the wet state. The appearance is consistent with that before modification, which is easy to use, and its light transmittance is not significantly different from that before modification. Because of the cross-linking effect of EDC/NHS, not only heparin is grafted to the surface of AM, but also the collagen of the AM can be cross-linked (Griffith et al., 1999; Vashist, 2012). Therefore, the mechanical strength of AM-HEP after rehydration is improved. The higher mechanical strength increases the operability of AM-HEP during use, which is a beneficial change (Long et al., 2014; Majumdar et al., 2018). At the same time, cross-linking will result in a tighter structure of collagen fibers, so the water content of AM-HEP has decreased (85%), but AM-HEP is still a material with a high water content. The water content of cornea is about 80%, so AM-HEP will not cause dryness and discomfort on the ocular surface (Patel et al., 2008). The microstructure of the material usually affects the performance of the material. Therefore, we observed the upper and lower surfaces of the modified AM. AM-HEP maintained the natural structure of AM. The collagen fibers and the unique D-period of collagen are clearly visible (Panwar et al., 2018). It showed that the modification process would not negatively affect the structure and performance of AM, and the higher mechanical strength and the introduction of heparin brought advantages to AM-HEP.

It is reported that heparin can electrostatically adsorb positively charged growth factors such as VEGF and TGF-β (Jo et al., 2015). From the results, AM-HEP can not only quickly absorb water, but also quickly adsorb EGF and achieve EGF sustained release *in vitro* (Figure 4). After confirming that the modified AM is not toxic to cells (Figure 5), in order to verify the biological activity of EGF released by AM-HEP@EGF, we coculture AM-HEP@EGF with CECs to observe the proliferation and migration of CECs (Figures 6, 7). We also set a commercialized BAM group for comparison. We observed that the cells cocultured with BAM and AM-HEP@EGF proliferate faster and can significantly promote cell migration under serum-free conditions. From the product information of the commercial BAM, we know that its EGF content is about 15 ng/g, which is lower than the EGF content of AM-HEP@EGF. This should be the reason of the fastest proliferation and migration ability of CECs, which were cocultured with AM-HEP@EGF. These results indicate that EGF released by AM-HEP@EGF can maintain function and activity and promote cell proliferation and migration. For the treatment of alkali burn, the proliferation and migration of epithelial cells affect the process of epithelialization, which is very necessary for the repair of ocular surface damage.

From the observation under clinical slit lamp for 14 days, the mice treated with AM-HEP@EGF exhibited differences of severity from 1 to 14 days (Figure 8). This indicated EGF was released from AM-HEP@EGF and acted on the ocular. The cornea treated with AM-HEP@EGF had the best transparency and epithelialization among all the groups. Compared to the control group from 1 to 14 days, the mice treated with AM exhibited increased efficacy but not as effective as AM-HEP@EGF. This may be because the content of growth factor on AM decreased after preparation process and long-time storage and led to unsatisfactory therapeutic effects (Pereira et al., 2016). Heparinization of AM can be an effective method to adsorb EGF and maintain the active of it. AM-HEP may also adsorb growth factors with positive charge such as VEGF, TGF-β, and nerve growth factor and apply to other application of tissue repair. Inflammatory factors play an important role in the healing process of corneal alkali burn. Injured corneal epithelial cells and stromal cells can release inflammatory factors such as TNF-α, IL-1β, and IL-6 after corneal alkali burn (Sotozono et al., 1997). The increase in these inflammatory factors may induce angiogenesis, inflammatory cell infiltration, and keratinocyte apoptosis. In the present study, although AM treatment after corneal alkali burn could down-regulate TNF-α, IL-1β, and IL-6 to some extent, AM-HEP@EGF treatment after corneal alkali burn was observed to have a better effect of down-regulating compared to AM treatment. These results suggest that AM-HEP@EGF treatments exhibited better anti-inflammatory effects compared to control and AM groups after corneal alkali burn.

## Conclusion

In this study, we have successfully prepared AM grafted with heparin, which can adsorb quickly, and sustained release EGF for treatment of ocular chemical burns. In general, introduction of heparin did not affect physical properties of AM and biocompatibility. AM-HEP had better EGF load capacity than AM. Faster corneal epithelialization was observed with the transplantation of AM-HEP@EGF *in vivo*. The corneas in the AM-HEP@EGF group have less inflammation and was more transparent than those in the control group. AM-HEP@EGF could significantly enhance the therapeutic effects. These results demonstrated that soaking heparinization AM into growth factor solution can be an effective method to supply growth factors to AM before use and solve the problem of insufficient growth factor content in AM after long-term storage. AM-HEP@EGF is exhibited to be a potent candidate for clinical application in corneal alkali burns treatment or accelerating epithelial wound healing. AM-HEP may be able to have more biological functions by adsorbing other growth factors and be applied to a wider range of tissue repair.

## Data Availability Statement

The raw data supporting the conclusions of this article will be made available by the authors, without undue reservation.

## Ethics Statement

The animal study was reviewed and approved by the Medicine Ethics Committee at Sun Yat-sen University.

## Author Contributions

XZh designed and performed the experiments and wrote the manuscript. XZu performed the *in vivo* experiments and wrote the Materials and Methods section of the manuscript. XZh and XZu contributed equally. JZ and BW helped with the cell culture. SL and YX helped with *in vivo* experiments and revised the manuscript. JY conceived and design the experiment and wrote the manuscript. All authors contributed to the article and approved the submitted version.

## Conflict of Interest

The authors declare that the research was conducted in the absence of any commercial or financial relationships that could be construed as a potential conflict of interest.
